# Supplementary material for: Assessing the influence of the health system on access to cervical cancer prevention, screening, and treatment services at public health centers in Addis Ababa, Ethiopia
Source: PLoS One. 2024 May 31;19(5):e0300152. doi: 10.1371/journal.pone.0300152 (PMC11142424; doi:10.1371/journal.pone.0300152)
Supplement: S1 File — (DOCX) [file pone.0300152.s001.docx]

**S1 File. Semi-structured questionnaire.**

1. **Establishing effective models of service delivery**

| **I. Facility services** |  | | |
| --- | --- | --- | --- |
| a. % of women (against the plan) reached with cervical cancer prevention awareness messages in the last 12 months. |  | | |
| b. % of girls (against the plan) fully vaccinated against cervical cancer at the age of 15 in the last 12 months. |  | | |
| c. % of women (against the plan) screened for cervical cancer in the last 12 months. |  | | |
| d. % of women (against the plan) with positive cervical screening were treated with cryotherapy in the last 12 months. |  | | |
| **II. Staff** | Yes | | No |
| a. Is the facility adequately staffed (against the health workforce plan)? | 1 | | 2 |
| b. If no (above), how many health workers (HWs) positions are vacant in the facility? | Specialty | | No. |
|  | a. Midwife | |  |
|  | b. Nurse | |  |
|  | c. Health officer | |  |
| c. The number of available staff who received training on screening and treatment in the last 24 months. | Specialty | | No. |
|  | a. Midwife | |  |
|  | b. Nurse | |  |
|  | c. Health officer | |  |
| **III. Financing** | Yes | | No |
| a. Is the facility adequately funded (against the annual budget) to be the center of care for cervical cancer patients? | 1 | | 2 |
| **IV. Provider** | Yes | | No |
| a. Do patients have any choice of provider? | 1 | | 2 |
| b. Do patients have a continuous relationship with an identified provider? | 1 | | 2 |
| **V. Health information systems** | Yes | | No |
| a. Is there an e-register for cervical cancer patients’ conditions? | 1 | | 2 |
| b. Is there patient call and recall systems (SMS/telephone) with automatic reminders for check-ups and screening appointments? | 1 | | 2 |
| **VI. Waiting times** |  | | |
| a. How long are the average waiting times in the facility? |  | | |
| b. Do waiting times meet the established standards? | Yes | | No |
|  | 1 | | 2 |
| **VII. Laboratory results** |  | | |
| a. What is the normal turnaround time (TAT) for receiving visual inspection with acetic acid (VIA) laboratory results? For receiving Pap smear laboratory results? | a. VIA |  | |
|  | b. Pap |  | |
| b. Does the TAT meet established standards for VIA? Also, for Pap smear? |  | Yes | No |
|  | a. VIA | 1 | 2 |
|  | b. Pap | 1 | 2 |
| **VIII. Referral system** | Yes | | No |
| a. Is the distance for referral to the hospital reasonable? | 1 | | 2 |
| b. Is there computers-e-referral? | 1 | | 2 |
| c. Do patient clinical records travel in either direction? | 1 | | 2 |
| d. Is there adequate transportation for referral services? | 1 | | 2 |

1. **Improving access to equipment, reagents, vaccines, and supplies**

| **Description of item** | **Available (Yes/No)** | |
| --- | --- | --- |
|  | Yes | No |
| a. Speculum | 1 | 2 |
| b. Cryotherapy machine with CO2 gas supply | 1 | 2 |
| c. Acetic acid | 1 | 2 |
| d. HPV vaccine | 1 | 2 |
| e. Is the following IEC/SBCC materials available? (Posters, Audio, Video, Flyer, Brochure, Other (specify), None) | 1 | 2 |
| f. Guideline for cervical cancer prevention and control | 1 | 2 |
| g. Referral forms | 1 | 2 |

1. **Linkage with the community and service users**

| **I. Training of health extension workers (HEWs)** | | Yes | No |
| --- | --- | --- | --- |
| a. Is the facility working with the HEWs for improving cervical cancer (CA) prevention and screening services? | | 1 | 2 |
| b. Are HEWs trained in cervical CA prevention and screening services? | | 1 | 2 |
| c. If yes (above), how many HEWs have been trained (out of the total number) in the last 24 months? Include 1) # of trained HEWs ----- 2) Total # of HEWs----- 3) %----- | |  | |
| **II. Roles of HEWs** |  |  | |
| (a) What are the areas of involvement of HEWs in cervical CA services?  **(MULTIPLE RESPONSES)** | a. Public Education | 1 | |
|  | b. Risk identification | 2 | |
|  | c. Screening/early detection | 3 | |
|  | d. HPV vaccination | 4 | |
|  | e. Referral to a health facility | 5 | |
|  | f. none | 6 | |
| (b) What are the public engagement areas for cervical CA services by HEWs?  **(MULTIPLE RESPONSES)** | a. Schools | 1 | |
|  | b. Community outreach | 2 | |
|  | c. Vulnerable populations | 3 | |
|  | d. Youth center | 4 | |
|  | e. Other | 5 | |
| **III. Prevention and screening services** |  |  | |
| What are the challenges to cervical CA public awareness, HPV vaccination, and screening?  **(MULTIPLE RESPONSES)** | a. Education | 1 | |
|  | b. Religious factors | 2 | |
|  | c. Stigma | 3 | |
|  | d. Language barrier | 4 | |
|  | e. Lack of space for screening | 5 | |
|  | f. Unavailability of SBCC/IEC materials | 6 | |
|  | g. Inadequate media coverage (TV, radio) | 7 | |
| **IV. Community linkage/referral** |  |  | |
| What are the major challenges in the community linkage or referral of the most at-risk women to the primary healthcare facility?  **(MULTIPLE RESPONSES)** | a. Transportation cost/allowance | 1 | |
|  | b. Distance to the facility | 2 | |
|  | c. Unavailability of referral forms | 3 | |
|  | d. Other | 4 | |
| What needs to be done to improve cervical cancer prevention and screening services? | a. Provide transportation allowance | 1 | |
|  | b. Shorten the waiting time | 2 | |
|  | c. Provide staff training | 3 | |
|  | d. Improve diagnostic capacity | 4 | |
|  | e. Ensure preference for a provider | 5 | |
|  | f. Spouse or partner support | 6 | |
|  | g. Promote media coverage | 7 | |
|  | h. Other, specify | 8 | |
