# Supplementary material for: Assessing the influence of the health system on access to cervical cancer prevention, screening, and treatment services at public health centers in Addis Ababa, Ethiopia
Source: PLoS One. 2024 May 31;19(5):e0300152. doi: 10.1371/journal.pone.0300152 (PMC11142424; doi:10.1371/journal.pone.0300152)
Supplement: S2 File — (DOCX) [file pone.0300152.s002.docx]

Table 1. Availability of equipment, vaccines, and supplies at health centers in Addis Ababa (n=51)

| Variables | Count | Percent |
| --- | --- | --- |
| Speculum |  |  |
| Available | 51 | 100.0 |
| Cryotherapy machine with CO_2_ gas supply |  |  |
| Available | 50 | 98.0 |
| Not available | 1 | 2.0 |
| Acetic acid |  |  |
| Available | 50 | 98.0 |
| Not available | 1 | 2.0 |
| HPV vaccine |  |  |
| Available | 28 | 54.9 |
| Not available | 23 | 45.1 |
| IEC/SBCC material (poster) |  |  |
| Available | 47 | 92.2 |
| Not available | 4 | 7.8 |
| IEC/SBCC material (audio) |  |  |
| Available | 6 | 11.8 |
| Not available | 45 | 88.2 |
| IEC/SBCC material (video) |  |  |
| Available | 5 | 9.8 |
| Not available | 46 | 90.2 |
| IEC/SBCC material (flyer) |  |  |
| Available | 39 | 76.5 |
| Not available | 12 | 23.5 |
| IEC/SBCC material (brochure) |  |  |
| Available | 44 | 86.3 |
| Not available | 7 | 13.7 |
| Guideline for cervical cancer prevention and control |  |  |
| Available | 51 | 100.0 |
| Referral forms |  |  |
| Available | 51 | 100.0 |

HPV- human papillomavirus; IEC - information, education, and communication; SBCC- social and behavior change communication

Table 2. Achievement (%) of cervical cancer services at health centers in Addis Ababa (n=51)

| Achievement* | Minimum | Maximum | Mean | Std. Deviation |
| --- | --- | --- | --- | --- |
| % of women reached with cervical cancer awareness messages. | 20 | 99 | 79.1 | 22.4 |
| % of girls fully vaccinated against cervical cancer at the age of 15. | 0 | 99 | 61.2 | 25.6 |
| % of women screened for cervical cancer. | 13 | 99 | 71.1 | 24.3 |
| % of women with positive cervical screening treated. | 1 | 99 | 79.8 | 33.3 |

*Achievement: Performance per plan for July 1, 2021 to June 30, 2022.

Table 3. Prevention, screening, and community linkage challenges, and improvement areas at health centers (n=51)

| Variables | Count | Percent |
| --- | --- | --- |
| Prevention and screening service challenge* |  |  |
| Education | 49 | 96.1 |
| Religious factors | 7 | 13.7 |
| Stigma | 9 | 17.6 |
| Language barrier | 5 | 9.8 |
| Lack of space for screening | 10 | 19.6 |
| Unavailability of SBCC/IEC materials | 6 | 11.8 |
| Inadequate media coverage (TV, radio) | 30 | 58.8 |
| Major challenges on community linkage/referral* |  |  |
| Transportation cost/allowance | 18 | 35.3 |
| Distance to the facility | 15 | 29.4 |
| Capacity of HEWs | 3 | 5.9 |
| Fear of procedure | 8 | 15.7 |
| Lack of awareness | 12 | 23.5 |
| Negligence | 5 | 9.8 |
| Areas of improvement in cervical cancer prevention and screening services* |  |  |
| Provide transportation allowance | 11 | 21.6 |
| Shorten waiting time | 8 | 15.7 |
| Provide staff training | 41 | 80.4 |
| Improve diagnostic capacity | 31 | 60.8 |
| Ensure preference for a provider | 13 | 25.5 |
| Spouse or partner support | 21 | 41.2 |
| Promote media coverage | 39 | 76.5 |
| Facilitate suitable room | 7 | 13.7 |
| Include routine HPV vaccine service | 4 | 7.8 |

*Participants who provided more than one response. IEC - information, education, and communication; SBCC- social and behavior change communication; HEWs- health extension workers; HPV- human papillomavirus.

Table 4. Key roles of health extension workers in the community (n=51)

| Variables | Count | Percent |
| --- | --- | --- |
| Areas of involvement of HEWs in the cervical CA services* |  |  |
| Public Education | 48 | 94.1 |
| Risk identification | 35 | 68.6 |
| Early detection | 29 | 56.9 |
| HPV vaccination | 15 | 29.4 |
| Referral to a health facility | 47 | 92.2 |
| Public engagement areas of HEWs for cervical CA services* |  |  |
| Schools | 36 | 70.6 |
| Community Outreach | 51 | 100.0 |
| Vulnerable populations | 30 | 58.8 |
| Youth center | 23 | 45.1 |
| House to house | 7 | 13.7 |

*Participants who provided more than one response. HEWs- health extension workers; CA – cancer

Table 5. Availability and training of staff in public health centers (n=51)

| Variables | Count | Percent |
| --- | --- | --- |
| Adequate staff available in the facility |  |  |
| Yes | 48 | 94.1 |
| No | 3 | 5.9 |
| Midwife staff received cervical cancer training* |  |  |
| Yes | 38 | 74.5 |
| No | 13 | 25.5 |
| Nurse staff received cervical cancer training* |  |  |
| Yes | 40 | 78.4 |
| No | 11 | 21.6 |
| Health officer staff received cervical cancer training* |  |  |
| Yes | 18 | 35.3 |
| No | 33 | 64.7 |
| Health extension workers received cervical cancer training* |  |  |
| Yes | 25 | 49.02 |
| No | 26 | 50.98 |

*In the 24 months preceding the study.

Table 6. Provider preference and information systems for cervical cancer in public health centers (n=51)

| Variables | Count | Percent |
| --- | --- | --- |
| Patients have any choice of provider |  |  |
| Yes | 33 | 64.7 |
| No | 18 | 35.3 |
| Patients have a continuous relationship with an identified provider |  |  |
| Yes | 41 | 80.4 |
| No | 10 | 19.6 |
| E-register for cervical cancer patients’ conditions in place |  |  |
| Yes | 7 | 13.7 |
| No | 44 | 86.3 |
| Patient call and recall systems (SMS/telephone) available in the facility |  |  |
| Yes | 39 | 76.5 |
| No | 12 | 23.5 |

Table 7. Turnaround time for laboratory tests and referral system in public health centers (n=51)

| Variables | Count | Percent |
| --- | --- | --- |
| TAT meets established standards for VIA test |  |  |
| Yes | 48 | 94.1 |
| No | 3 | 5.9 |
| TAT meets established standards for Pap smear test |  |  |
| Yes | 2 | 3.9 |
| No | 49 | 96.1 |
| Reasonable distance for referral to the hospital (less than 10 km) |  |  |
| Yes | 39 | 76.5 |
| No | 12 | 23.5 |
| Computers-e-referral in place |  |  |
| Yes | 0 | 0.0 |
| No | 51 | 100.0 |
| Patient clinical records travel in either direction |  |  |
| Yes | 25 | 49.0 |
| No | 26 | 51.0 |
| Adequate transportation available for referral services |  |  |
| Yes | 18 | 35.3 |
| No | 33 | 64.7 |

TAT – turnaround time; VIA- visual inspection with acetic acid; Pap- Papanicolaou


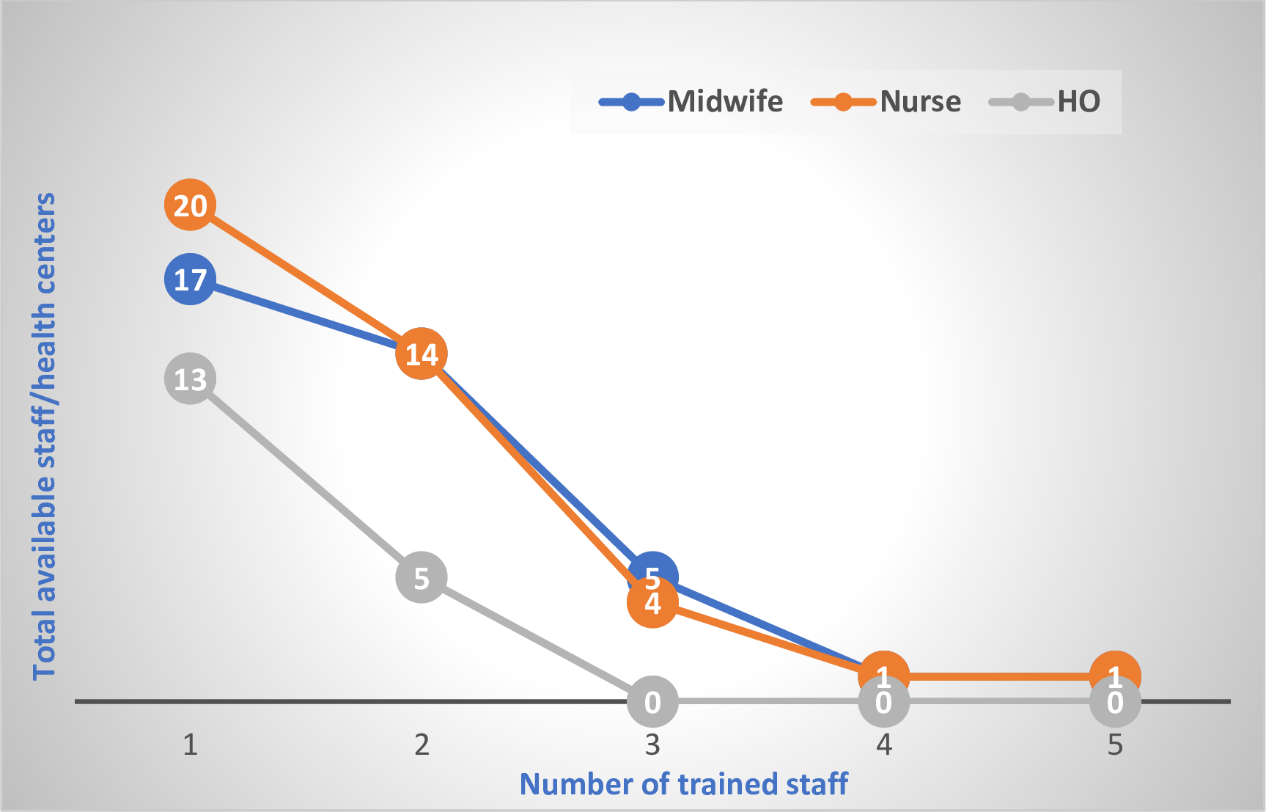


Fig 1. Trained staff in public health centers in 24 months preceding the study (n=51)


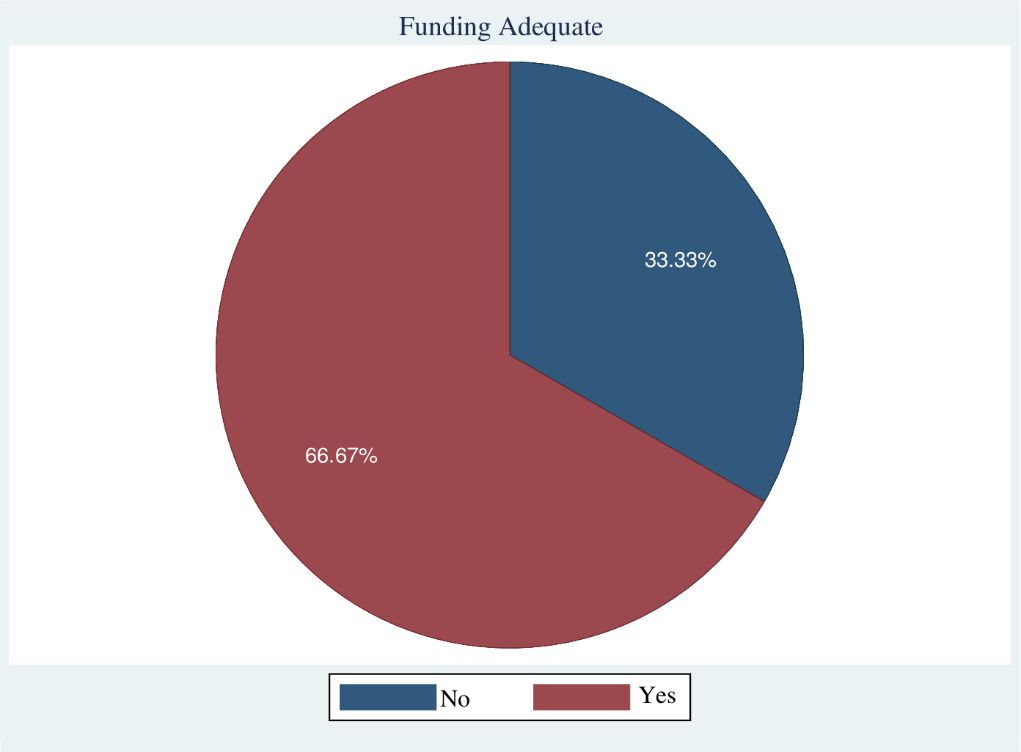


Fig 2. Percentage of the adequacy of cervical cancer funds in public health centers (n=51)


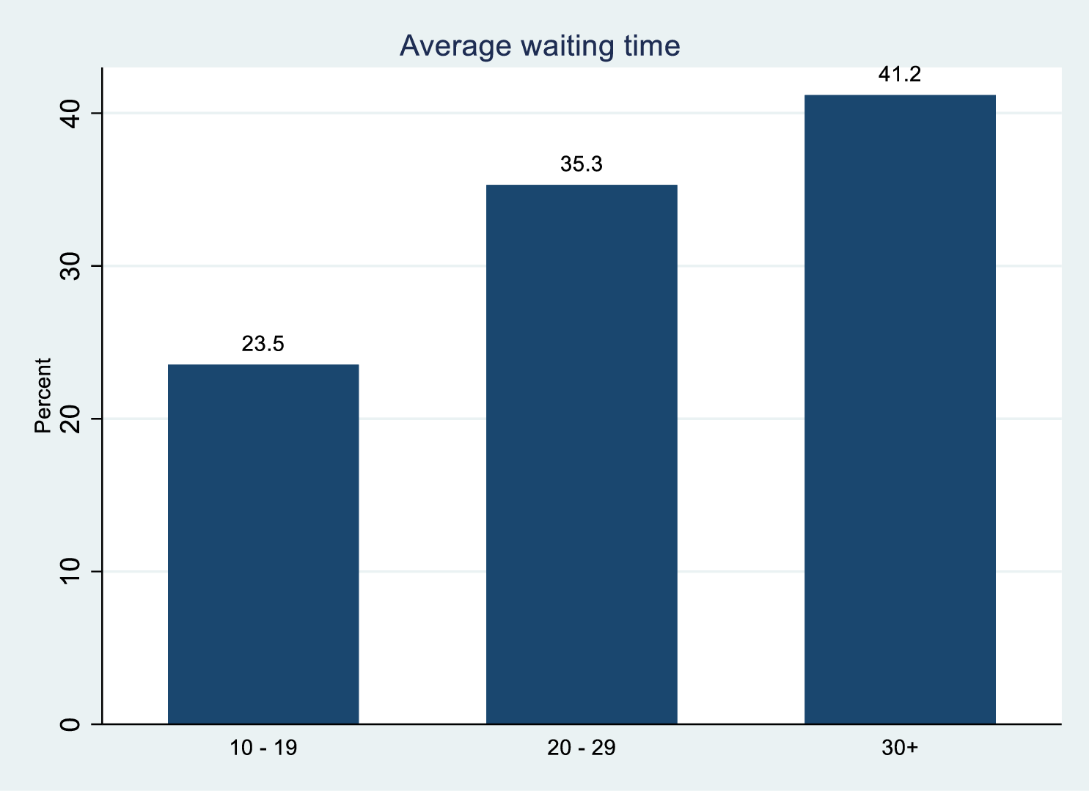


Fig 3. Waiting time (in minutes) for cervical cancer services in public health centers (n=51)
