# Supplementary material for: Assessing the influence of the health system on access to cervical cancer prevention, screening, and treatment services at public health centers in Addis Ababa, Ethiopia
Source: PLoS One. 2024 May 31;19(5):e0300152. doi: 10.1371/journal.pone.0300152 (PMC11142424; doi:10.1371/journal.pone.0300152)
Supplement: Attachment — Submitted filename: Response to Reviewers.docx [file pone.0300152.s004.docx]

**Manuscript Number: PONE-D-23-08979**

**Response to Reviewers**

**Response to the Academic Editor**

| **Comment** | **Response** | **Revisions made in the manuscript** | **Page** | **Line #** |
| --- | --- | --- | --- | --- |
| There are major flaws in grammar and language throughout this paper. In the revised version, try to address this. Authors are highly recommended to get editions services of this manuscript with experts, to have a qualified scientific paper. | We accept your comment. | We have now tried to revise and improve the grammar and language throughout the manuscript. | 1-20 | 11-441 |
| The abstract section of the paper should clearly show the rationale for this paper, and how the proposed aim was answered. But, these issues are shallow and lack clarity. The method section in the main body is not also clearer and doesn't help with replication. Therefore, the authors are suggested to revise this section and better if the methods are re-organized in the following sections: study settings and period, Study design and participants, data collection, study variables, data quality control, data processing and analysis, ethical consideration | We accept your recommendation. | The Abstract section is now revised to clearly show the rationale for this paper, and how the proposed research question was answered.  The Methods section is revised to help with the replication and re-arranged as follows:   - Study settings and period - Study design and participants - Sample size and sampling procedure - Data collection procedure - Study variables - Data quality control - Data processing and analysis - Ethical consideration | 1  4-9 | 10-37  89-207 |
| In the topics and aim of the paper, key terms like equity, quality of cervical cancer services, and the like are described. However, the authors didn't adequately define these terms in any section of the paper, not described the measurement methods employed, and displayed the measured results. Though two frameworks are employed in the current paper, how this framework is employed to guide the measurements lacks clarity. Please provide explanations for this, and as well address these issues within the manuscript. | We accept your comment.  The explanations or operational definitions of key terms such as “equity” and “quality” of cervical cancer services are now included in the introduction section. This is also included in the measurement methods and in the results section. The two frameworks are now further explained to guide the measurement methods. However, “quality” is removed from the title and the focus will be on other health system factors (target achievement, timeliness, availability, patient-centeredness, and equity) that are influencing the provision of cervical cancer services. | Quality healthcare in Ethiopia calls for comprehensive care that is timely, affordable, efficient with its use of resources, effective, safe, and patient-centered. Similarly, in this study, a "quality cervical cancer service" refers to how well the desired health outcomes (target achievement, timeliness, patient-centeredness, availability, and equity) are improved by providing women with services for prevention, screening, and treatment. While "equity" seeks to provide high-quality healthcare services to all people without distinction based on their geographical location, gender, income, or disabilities.  The two frameworks are further explained to guide the measurement. Moreover, this is included in the measurement methods and displayed in the measured results. | 3  3-4  4  3  6-7  9-13 | 81-83  83-86  86-88  73-81  141-165  208-267 |
| Make sure that the manuscript has addressed the journal’s requirements in terms of contents, particularly after the conclusions section of the paper. Refer to the journal guideline and correct this part. | We accept your recommendation. We used square brackets in our citations. This will be applied in the revised manuscript as well. | We have followed the journal guidelines requirements and particularly corrected the contents after the conclusion section including the “Author’s contributions” part. | 21 | 442-451 |
| Change the citation formats to square brackets (e.g., [1]). |  | All the citations are now in square brackets. | 2-20 | 39-434 |
| Adequately discuss the present study findings with that of literature in other settings (out of Ethiopia). Discuss the strength and limitations of the study, focusing on the methods used, and have a separate section to discuss the policy and practice implications of the study. | We are in agreement with your comment. | We have included some recent findings from other parts of the world (outside Ethiopia).  We have discussed the “strengths and limitations” of the study, focusing on the methods used. We have also provided a separate section on the “policy and practice implications of the study.” | 14  15  17,20  17-18  18-19 | 288  314  356, 421  357-382  383-402 |

**Response to Reviewer # 1**

| **Manuscript section** | **Comment** | **Response** | **Revisions made in the manuscript** | **Page** | **Line #** |
| --- | --- | --- | --- | --- | --- |
| Title | 1. Either modify the title or re-do the analysis because there is no result to talk about the quality of service. Also, there is no strongly concluded point about equity to the service user. | We agree with your observation and revised the title by removing the “quality” and “equity”. The conclusion about equity to the service users is now revised. | The new title is “Health system factors affecting access to cervical cancer prevention, screening, and treatment services at public health centers in Addis Ababa, Ethiopia.” | 1  16  19 | 1-3  330-346  404-408 |
| Introduction | 2. The 150,000 new cases that Ethiopia reports is not clear. Line 46 | We accept this comment and now revised the information. | Each year, hospital data in Ethiopia revealed over 150,000 new cases of cancer, which account for 4% of all deaths | 2 | 49-50 |
|  | 3. Font Color of lines 56-59 is different: a light blue. Better to make it similar throughout the document. The sentence is direct copy and paste. Rephrase | We agree with your comment. The Font Color of lines 56-59 is now made black. Moreover, the Font Color is black throughout the manuscript.  The sentence is re-phrased. | In order to prepare the way for the eradication of cervical cancer in the twenty-first century, WHO urges nations to achieve the 90-70-90 cervical cancer targets by 2030. At the age of 15, 90% of girls take the HPV vaccine in full, 70% of women undergo cervical screening at the age of 35 and 45, and 90% of women with precancerous lesions or invasive cancer receive treatment. | 2-3 | 58-62 |
|  | 4. I was expecting to read what “quality cervical cancer services” are mean in the introduction, particularly in Ethiopia. How did it become your concern? Any other previous evidence on the case and what is the gap? | We have now provided what “quality” cervical cancer services mean in the Ethiopian context in the “Introduction”. We considered “quality” to assess the extent to which the existing cervical cancer services (HPV vaccination, screening, and treatment) provided to women improve the desired health outputs in public health facilities. To achieve this, a health system is required to consider the six dimensions of quality as defined by WHO (2006) and the Ethiopian context which include the care is 1) effective, 2) efficient, 3) accessible, 4) acceptable/patient-centered, 5) equitable, and 6) safe.  There was no other prior evidence on this. Hence, there was a gap in understanding the health system inputs and outputs/processes for the provision of quality cervical cancer prevention and control services at the primary healthcare (health center) level. | In Ethiopia, "Quality cervical cancer services" refer to how well the desired health outcomes (target achievement, timeliness, patient-centeredness, availability, and equity) are improved by providing women with services for prevention, screening, and treatment. | 3-4 | 83-86 |
| Materials and Methods | 1. The method needs rearrangement. For example, why data collection method under the session “Study design and period”? | We accepted this comment and made a rearrangement of the methods section. The data collection method is removed from “Study Design and Period”, and put under “Data collection procedure.” | The Methods section is reorganized as follows:   - Study settings and period - Study design and participants - Sample size and sampling procedure - Data collection procedure - Study variables - Data quality control - Data processing and analysis - Ethical consideration | 4-9 | 89-207 |
|  | 2. Organization of Ethiopian health service delivery system line 93-95 needs reference. | We have now provided the reference: Ethiopian Health Sector Development Plan (2010-2015). | Reference included: “Ethiopian Health Sector Development Plan (2010-2015).” | 4 | 94 |
|  | 3. It is described how the health facilities were sampled and selected. How about the service providers? Who were they? How were they selected? It should not be indicated under the session “Data collection procedure and data analysis”. | We have now provided how the service providers were sampled and selected. This information is now included under “Sample size and sampling procedure.” | After considering the "cervical cancer focal person position" in health centers, 51 healthcare workers (nurses, midwives, and health officers) were chosen for face-to-face interviews using a semi-structured questionnaire. The focal persons are individuals responsible for managing or coordinating cervical cancer services. | 5 | 116-121 |
|  | 4. The data collection procedure is not indicated well. Face-to-face interview? FGD? Checklist data extraction??? | We have now indicated this information under the “Data collection procedure.” | The data collection procedure included two components:  1. Face-to-face interviews: After approval of the study by the ethics committee, the data were collected from the 51 health centers through face-to-face interviews with cervical cancer focal persons by five trained data collectors (public health experts) using an Open Data Kit (ODK) tool installed with an English language semi-structured questionnaire. The semi-structured questionnaire was read out to cervical cancer focal persons and explained (translated) in Amharic (local language) to avoid any misunderstanding by the cervical cancer focal persons. The survey was focused on health system performance “inputs” (providers, equipment, tests and vaccines, financing, and health information systems), and “outputs” (target achievement of services, availability of screening options, timeliness of services, patient-centeredness, and equity) of cervical cancer services.  2. Secondary data review: The second part of the data collection procedure involved a review of existing data related to cervical cancer. The checklist was used for the data extraction by examining records from the cervical cancer register, the extended program on immunization (EPI) register, and administrative reports. The cervical cancer register provided information on the number of women who were screened for cervical cancer and who received treatment. The EPI registration was investigated to ascertain the number of girls who received the HPV vaccine. Administrative reports, on the other hand, provided information on public awareness messages related to cervical cancer. | 5-6 | 122-140 |
|  | 5. Avoid unnecessary repetitions. For example, the study period on lines 91 and 118. | We agree with this observation. We have now avoided repetition. | We removed “The study was conducted between July 2022 and August 2022 in Addis Ababa” from “Study Design and Period”, and “Data Collection Procedure and Data Analysis”. This information is now put under “Study Settings and Period”. | 4 | 99 |
|  | 6. The data analysis procedure lacks a detailed explanation. Is it not important to explain the used analysis method, and cutoff points,…the analysis is rough and do not fully support the study aims. | We agree with this comment. We have now provided a revised and detailed data analysis procedure to fully support the study aims under “Data Processing and Analysis.” | To make sure that the data was correct, trustworthy, and prepared for descriptive analysis, the following approach was employed. Data auditing was done before data collection was finished to make sure the required sample size is kept. This process included checking the collected data for accuracy, consistency, and completeness. Any discrepancies or missing data points were fixed. The data was exported to Excel CSV format after the data auditing procedure was finished. In order to further clean and analyze the data, SPSS Version 26 was employed. At this step, extra verifications and checks were done on the data to make sure it is accurate and consistent. This made it possible to find mistakes and fix them, deal with missing values, and recode variable replies so that they are in the correct format. The dataset was cleaned and then made ready for additional analysis. In this step, certain variables of interest were chosen and the data was formatted in a way that is suitable for analysis. Descriptive statistics was used to summarize the data. Graphs and tables were used to summarize the percentage and attributes of the cervical cancer focal persons' responses, as well as the secondary data extracted from health centers' cervical cancer register, EPI register, and administrative reports. | 8-9 | 184-199 |
|  | 7. It is important to describe measurements in this study. For example,  o How the fulfillment of the input was measured? Was it the summation of all the available inputs or any of them? Was 100% input expected?  o What indicators were used to measure (effectiveness, availability, timeliness, and patient-centeredness): the Outputs?  o How was equity of cervical cancer services measured? When did you say there is equity in the services?  o What is quality service as per your study? | We have now provided this information on:   - How the fulfillment of the input was measured? - What indicators are used to measure the outputs? - How was the equity of cervical cancer services measured? - What does quality service in this study mean? | The following measurements were used in this study. These are put under the “Study Variables” section.   - The health system “inputs” were measured considering the availability of cervical cancer personnel (human resources), screening devices, tests, treatment equipment, and HPV vaccine (physical/infrastructure), health information system (information), and funding, user fees, and transportation fees (financial). The study considered the summation of all the available inputs (without 100% expectations). - The measurement of health system “output” indicators was done as follows 1) target achievement- the HPV vaccination, cervical cancer screening and treatment services achievement (performance per annual plan) was measured against progress toward meeting the WHO 90-70-90 targets by 2030; 2) availability- it measured the availability of screening/testing options, 3) timeliness- the screening and treatment services were measured against the standards set by the national guidelines, and 4) patient-centeredness- it measured whether services were given with patient preferences. - Equity was measured based on whether there are cervical cancer prevention, screening, and treatment services at 51 randomly selected health facilities in 11 sub-cities of Addis Ababa without variation due to socioeconomic status, ability to pay, and geographical location. - In this study, “quality service” means the extent to which HPV vaccination of girls, dissemination of public awareness messages, screening, and treatment services provided to women improve the desired health outcomes (target achievement, timeliness, patient-centeredness, availability, and equity). | 6-7  7  7  3-4 | 146-153  154-165  163-165  83-86 |
| Results | 1. What is the importance of writing in different colors again? Line 134-139 | We agree with your observation. The Font Color is black throughout the manuscript. | The font color is black. | 1-24 | 11-567 |
|  | 2. You have no data extraction method. How was the data for the information provided in Table 3 were obtained? | We have now provided the data extraction method for the information provided in Table 3. | A checklist (for percentage achievement of public awareness, HPV vaccination, screening, and treatment) was used for the data extraction by examining records from the cervical cancer register, the extended program on immunization (EPI) register, and administrative reports. The cervical cancer register provided information on the number of women who were screened for cervical cancer and who received treatment. The EPI registration was investigated to ascertain the number of girls who received the HPV vaccine. Administrative reports, on the other hand, provided information on public awareness messages related to cervical cancer. | 6 | 134-140 |
| Discussion | 1. Lines 192-194: “The study revealed that the prevailing health system challenges at the primary healthcare level had negatively impacted the quality of cervical cancer 194 services.” How can you say it affects the quality without showing the status of the quality of the service? | We accept your comment and removed the “quality” issue including from the title of the study. Our intention was to assess the quality of cervical cancer services as defined by WHO (2006) and the Ethiopian context (timeliness, effectiveness, patient-centeredness, availability, and equity). However, we agree that the focus should be on the health system factors affecting cervical cancer services rather than the quality of services. |  |  |  |
|  | 2. Ideas in lines 197-216 seem taken from literature; how does the idea on line 217 connect with it? Which findings are being compared? | The information in lines 197-200 was obtained from the Ministry of Health documents. While the findings from 200-216 are from our study. Routine HPV immunization was a challenge due to the unavailability/irregular availability and high loss of the vaccine. Pap smear testing (screening) was a challenge due to its unavailability at health centers. Hence, all these factors connect with line 217, and an additional reference is provided to support this. This tries to discuss the findings of the study focusing on the availability of vaccines, tests, and equipment for screening of cervical cancer (comparing the findings with the previous studies). | Revisions are made and an additional reference is provided to support this (Ministry of Health and Ethiopian Public Health Institute. Ethiopia-Service-Availability-and-Rediness-Assessment-SARA-report; 2018). | 14-15 | 298-315 |
|  | 3. Line 233-234, client report. Where did you get it? You have no client involvement or data extraction in the data collection method. | We agree with your observation. We removed the “clients also reported waiting too long before receiving service at some facilities.” We have now provided the “data extraction method.’’ | A checklist (for percentage achievement of public awareness, HPV vaccination, screening, and treatment) was used for the data extraction by examining records from the cervical cancer register, the extended program on immunization (EPI) register, and administrative reports. The cervical cancer register provided information on the number of women who were screened for cervical cancer and who received treatment. The EPI registration was investigated to ascertain the number of girls who received the HPV vaccine. Administrative reports, on the other hand, provided information on public awareness messages related to cervical cancer. | 6 | 134-140 |
|  | 4. In general, I recommend doing the discussion ideally under sub-headings of input, output, and equity in access to cervical cancer screening. It is a copy of the result. Make it short and discuss using guidelines on recommended minimum importance. | We agree with your recommendation. We shall stick to the guidelines and make the discussion succinct. | The discussion is reorganized into the sub-headings of input, output, equity, and areas of improvement to access cervical cancer prevention, screening, and treatment services. | 13-17 | 268-356 |
| Conclusions | Lines 312-313: do you have data on the poor engagement of school and drop-out girls? | There was no data on the poor engagement of school and drop-out girls in lines 312-313. This sentence is now removed. |  |  |  |
|  | Similarly, in lines 316-317; do you have information on “HPV DNA and Pap smear testing attract more women for screening”? | Yes, there were previous studies that proposed the vaginal self-sampling of HPV testing including in high-burden and resource-limited settings. We have included a couple of studies that are supporting HPV DNA testing. However, there was no supporting study on Pap smear testing showing that it attracts more women for screening. | Studies supporting HPV DNA testing are included: In Ethiopia (Bogale AL et al., 2022), and Central America (Holme F, 2020). | 20 | 421 |
|  |  the conclusion should be done depending only on the data that can be accessed in this manuscript. It seems discussion. | We accept your comment. We have now revised the conclusion. | The conclusion is revised based on the data accessed in this manuscript. | 19-20 | 403-434 |

**Response to Reviewer # 2**

| **Manuscript section** | **Comments** | **Response** | **Revisions made in the manuscript** | **Page** | **Line #** |
| --- | --- | --- | --- | --- | --- |
| Abstract | Lines 19-20: be consistent in the use of terms (women vs girls) use either of the two throughout your documents, again in the use of health center and health facilities | We accept your comment and ensure that women or girls are used in the right place in the manuscript. However, for lines 19-20, public education, screening, and treatment are mainly provided to women while HPV vaccination is for girls. On the other hand, since health facilities include health posts, health centers, and hospitals, we propose to use “health centers” throughout the document. | We used girls for HPV immunization, and women for public education, screening, and treatment of cervical cancer throughout the manuscript.  Health centers were used rather than health facilities to describe the study sites. | 1-20  1-20 | 11-441  11-441 |
| Introduction | 1. Line 39: add references at the end of the paragraph. | We agree with your recommendation. We revised the sentence and included the reference: WHO. Global strategy to accelerate the elimination of cervical cancer as a public health problem (2020). | Though cervical cancer is a preventable disease and curable (with early detection, timely diagnostic follow-up, and effective treatment) it continues to be a public health problem resulting in the premature death of women mainly in low-income countries globally [1]. | 2 | 41 |
|  | 2. Line 56-59: be alert while taking documents from other sources and I will recommend you to re-write again. | We agree with your comment and the sentence is now rephrased. | In order to prepare the way for the eradication of cervical cancer in the twenty-first century, WHO urges nations to achieve the 90-70-90 cervical cancer targets by 2030. At the age of 15, 90% of girls take the HPV vaccine in full, 70% of women undergo cervical screening at the age of 35 and 45, and 90% of women with precancerous lesions or invasive cancer receive treatment. | 2-3 | 58-62 |
| Methods | 3. Lines 92 – 101: what is your study population: healthcare providers or health centers?? | The study population is health centers. | The study population was 90 public health centers actively providing cervical cancer prevention, screening, and treatment services in Addis Ababa. | 4 | 103-104 |
|  | 4. A proportionate number of 51 facilities were randomly selected using Microsoft Excel out of a total of 90 public health centers actively providing cervical cancer services. How do you select healthcare providers from each health center? Do you think equal numbers of healthcare providers were in each HC? | We have now included how the healthcare providers from each health center were selected. This will come under “Sample size and sampling procedure.” | After considering the "cervical cancer focal person position" in the health centers, 51 healthcare workers (nurses, midwives, and health officers) were chosen for face-to-face interviews using a semi-structured questionnaire. The focal persons were individuals responsible for managing or coordinating cervical cancer services. However, the number of cervical cancer service providers was different from a health center to a health center. | 5 | 116-121 |
|  | 5. Line 113-114: you said cervical cancer service providers in facilities (11 nurses, 10 midwives, 4 health officers, and 26 cervical cancer focal persons) were approached  What is your justification to select nurses, midwifery, and health officers? Why not used cervical cancer focal persons throughout all health facilities? | We agree with your comment. The justification for the selection of cervical cancer service providers in facilities (nurse, midwife, or health officer) was whether the person holds the focal person position. So, we have used cervical cancer focal persons throughout the survey in all health centers. | The service providers were selected considering the cervical cancer focal person position in the health facility. The focal persons were nurses, midwives, or health officers. The focal persons are individuals responsible for managing or coordinating cervical cancer services. This information is included under “Sample size and sampling procedure.” | 5 | 116-121 |
|  | 6. Line 114: add health be for the word facilities, again be consistent in the use of health centers and facilities (I recommend using health facilities throughout your documents). | We agree with your recommendation (consistency in using words). However, health facilities include health posts, health centers, and hospitals. Thus, we propose to use “health centers” throughout the document. |  | 1-20 | 11-441 |
|  | 7. You need to provide more details on the study recruitment process and data collection.  o How exactly were the healthcare providers recruited from 51 health centers? | We have now included:  How the healthcare providers from the 51 health centers were recruited.  We have now p healt | The healthcare providers were recruited considering the cervical cancer focal person position throughout all health centers. The focal persons are individuals responsible for managing or coordinating cervical cancer services. | 5 | 116-121 |
|  | - How do you select 51 health centers from 11 sub-cities? Clearly shown in sampling procedures. | How the 51 health centers from 11 sub-cities were selected. | A proportionate number of 51 health centers were randomly selected using Microsoft Excel out of a total of 90 public health centers actively providing cervical cancer services in 11 sub-cities. The list was obtained from the Addis Ababa City Administration Health Bureau. | 5 | 113-116 |
|  | - Where was the data collected, and for how long? Etc. etc. | The data was collected in Addis Ababa from July 2022 to August 2022. We have included this under “Study settings and period.” | The data was collected in Addis Ababa from July 2022 to August 2022. | 4 | 99 |
|  | - Since these healthcare providers were not interviewed in the local language even if they were understanding English? What measures were in place to ensure that the translation process, did not affect the content of what was said? | We have now provided the measures taken to ensure the translation process of English to the local language (Amharic). | The semi-structured questionnaire was read out to cervical cancer focal persons and explained (translated) in Amharic (local language) to avoid any misunderstanding. | 5-6 | 127-128 |
|  | - Lines 120: change ethical consideration to Ethics approval and consent to participate. I recommend you strictly follow journal guidelines. | We have checked the journal guidelines, and “Ethical Consideration” was used throughout PLOS ONE published papers. The academic editor was also referring to “Ethical Consideration” | We have kept the “Ethical Consideration” sub-heading under “Materials and Methods.” | 9 | 200 |
|  | - Lines 125-126: All methods were performed in accordance with the relevant guidelines and regulations (Declaration of Helsinki). Do the PLoS One guidelines follow such terms? | We accept your comment. We have now removed the Declaration of Helsinki. | We have included “the cervical cancer focal persons were briefed on the purpose and objective of the research” under “Ethical Consideration.” | 9 | 205-206 |
|  | - On line 174: table 3: I think you have used document review for data retrieval, my concern is what are your data collection methods (state clearly). | We have now provided the data collection method for Table 3. This will come under the “Data collection procedure.” | A checklist (for percentage achievement of public awareness, HPV vaccination, screening, and treatment) was used for the data extraction by examining records from the cervical cancer register, the extended program on immunization (EPI) register, and administrative reports. The cervical cancer register provided information on the number of women who were screened for cervical cancer and who received treatment. The EPI registration was investigated to ascertain the number of girls who received the HPV vaccine. Administrative reports, on the other hand, provided information on public awareness messages related to cervical cancer. | 6 | 134-140 |
| Results | General comments  If the socio-demographic variables were in your tools, better to add the description of socio-demographic characteristics of health care providers (age, sex, profession, etc) | The research tool did not have the socio-demographic variables. Hence, the manuscript does not have a description of the socio-demographic characteristics of healthcare providers (cervical cancer focal persons). |  |  |  |
|  | 8. Lines 130-131: your description were 20, 17, and 13 health facilities. The total was 50 versus 51 health facilities. | This is to show the available minimum number of cervical cancer-trained staff (nurse, midwife, or health officer) in health centers. So, the trained number of staff does not necessarily sum up to 51 health facilities. | There was at least one nurse, one midwife, and one health officer trained in cervical cancer services in 20, 17, and 13 health centers, respectively. | 9 | 211-212 |
|  | 9. Lines 131-132: Rephrase the sentence at lines 131 and 132 to make correct meaning.  The recommendation is to minimize use respectively when your list of activities were more than three. In 40 (78.4%), 38 (74.5%), 18 132 (35.3%), and 25 (49%) facilities had nurses, midwives, health officers, and health extension 133 workers (HEWs) who received cervical cancer training, respectively  You can write as 40 (78.4%) nurses, 38 (74.5%) midwives, 18 (35.3%) health officers, and 25 (49%) health extension workers (HEWs) received cervical cancer training | We agree with your comment. We have now included the revised write-up per your recommendation. | Among the 51 health centers surveyed, the staff who received cervical cancer training in the 24 months preceding the study were 40 (78.4%) nurses, 38 (74.5%) midwives, 18 (35.3%) health officers, and 25 (49%) health extension workers (HEWs). | 9 | 212-214 |
|  | 10. Line: 142: table 1: revised all tables based on journal guidelines formats | We accept your comment and ensured that the tables are inserted per the journal guidelines format. | We revised and cited tables in ascending numeric order upon the first appearance in the manuscript file. | 10  11  12-13 | 226-227  241-242  265-267 |
|  | 11. Lines 161-165: this whole text descriptions were not in line with the tables and need to be corrected | We accept your comment and corrected the text descriptions in lines 161-165 by putting the exact figures. |  | 12 | 260-264 |
|  | 12. Lines 170: How do you measure the effectiveness of services? needs operational definition in the methods part | We have now preferred to include “target achievement” than “effectiveness”. Hence, we have provided the operational definition of the “target achievement” of cervical cancer prevention and control. | Target achievement: the HPV vaccination, cervical cancer screening, and treatment services achievement (performance per the annual plan) was measured against progress toward meeting the WHO 90-70-90 targets. | 7 | 155-159 |
| Discussion and conclusions | Detail and clear description of each section | We have now provided a detailed and clear description of the discussion and conclusions parts. | A detailed and clear description of the following sub-headings is provided in the discussion section: inputs, outputs, equity, and areas of improvement to access to cervical cancer prevention, screening, and treatment services. The conclusions section is revised based on the accessed data at the health centers. | 13-20 | 268-434 |
