# Supplementary material for: Assessing the influence of the health system on access to cervical cancer prevention, screening, and treatment services at public health centers in Addis Ababa, Ethiopia
Source: PLoS One. 2024 May 31;19(5):e0300152. doi: 10.1371/journal.pone.0300152 (PMC11142424; doi:10.1371/journal.pone.0300152)
Supplement: Attachment — Submitted filename: The manuscript overall would benefit from some significant editing services.docx [file pone.0300152.s005.docx]

1. The manuscript overall would benefit from some significant editing services; the grammatical mistakes make it difficult to concentrate on the content of the manuscript.
2. Conclusion in the abstract section seems to be refined as it doesn’t fit with the aim the study line 31-33
3. As per your introduction of study, the study seems that you conducted associated factors specifically line 72-73.
4. What is the importance of these statement ” It borrows from a framework proposed by Kruk and Freedman that links health system “inputs” (financial, human, physical, and information systems) to “outputs” such as target achievement of the HPV vaccination, cervical cancer screening, and treatment services, availability of screening options, timeliness of screening and treatment services, patient-centeredness, and equitability to screening and treatment services including for disadvantaged groups. It also draws on the conceptual framework for the modified social-ecological model that looks at the factors from the individual level, health facility level, and wider health system level perspective, and their linkage to delay in reaching a health facility and delay in receiving appropriate care “ rather it’s better to show gaps clearly.
5. Under methods section line 99 clearly put the period of data collection period
6. Clearly mention your study participants
7. Specify type of consent you took line 206
