# Supplementary material for: Assessing the influence of the health system on access to cervical cancer prevention, screening, and treatment services at public health centers in Addis Ababa, Ethiopia
Source: PLoS One. 2024 May 31;19(5):e0300152. doi: 10.1371/journal.pone.0300152 (PMC11142424; doi:10.1371/journal.pone.0300152)
Supplement: Attachment — Submitted filename: Response to reviewers.docx [file pone.0300152.s006.docx]

**Manuscript Number: PONE-D-23-08979**

**Response to Reviewers**

| **Comment** | **Response** | **Revisions made in the manuscript** | **Page** | **Line #** |
| --- | --- | --- | --- | --- |
| 1. The manuscript overall would benefit from some significant editing services; the grammatical mistakes make it difficult to concentrate on the content of the manuscript. | - We agree with your recommendation. We made some editions focusing on identifying and making corrections to grammatical mistakes. The manuscript is now written in Standard English to make it more clear, correct, and unambiguous for understanding its content. | - Throughout the manuscript. - Reference # 41 is included: Atnafu T, Daka DW, Debela TF, Ergiba MS. Women’s Satisfaction with Cervical Cancer Screening Services and Associated Factors in Maternal Health Clinics of Jimma Town Public Health Facilities, Southwest Ethiopia. CMAR. 2021; 13:7685–96. | 1-19  23 | 1-410  542-544 |
| 2. Conclusion in the abstract section seems to be refined as it doesn’t fit with the aim the study line 31-33 | - We agree with your comment. We have revised the conclusion section to reflect the study objectives. Additionally, reviewer #2 stated that the conclusion is too long and needs revision. Hence, we made it short with the necessary revisions. | - The conclusion in the Abstract section is revised. - The “conclusions section” is revised. | 1  19 | 31-34  394-410 |
| 3. As per your introduction of study, the study seems that you conducted associated factors specifically line 72-73. | - We respect your comment and revised the statement specifically in lines 72-73. The study intended to describe the health system issues of cervical cancer services. | - Our study aimed to assess the health system's influence on access to cervical cancer prevention, screening, and treatment services at public health centers in Addis Ababa. | 3 | 71-72 |
| 4. What is the importance of these statement ” It borrows from a framework proposed by Kruk and Freedman that links health system “inputs” (financial, human, physical, and information systems) to “outputs” such as target achievement of the HPV vaccination, cervical cancer screening, and treatment services, availability of screening options, timeliness of screening and treatment services, patient-centeredness, and equitability to screening and treatment services including for disadvantaged groups. It also draws on the conceptual framework for the modified social-ecological model that looks at the factors from the individual level, health facility level, and wider health system level perspective, and their linkage to delay in reaching a health facility and delay in receiving appropriate care “rather it’s better to show gaps clearly. | - We accept your comment and removed the unnecessary paragraph as this is included in the “study variables” section. | - The study took into account components of the earlier conceptual frameworks to examine the health system's "inputs" and "outputs" related to services for cervical cancer [16,17] | 3 | 72-74 |
| 5. Under methods section line 99 clearly put the period of data collection period | - We agree with your recommendation. Now we put the exact dates of the data collection. | - The study's data collection period was from July 15, 2022, until August 31, 2022. | 4 | 91-92 |
| 6. Clearly mention your study participants | - Per your suggestion, we revised the participants section. | - Study participants comprised 51 cervical cancer focal persons appointed by the 51 health centers chosen for the study. Nurses, midwives, and health officers who supervise, manage, and coordinate cervical cancer services are the focal individuals in Addis Ababa's health center settings. | 4 | 97-100 |
| 7. Specify type of consent you took line 206 | - We concur with your comment. We revised this statement. | - The written informed consent was obtained from respondents before the interview. | 9 | 195 |
